# Electrochemical Polymerization of Hydroquinone on Graphite Felt as a Pseudocapacitive Material for Application in a Microbial Fuel Cell

**DOI:** 10.3390/polym9060220

**Published:** 2017-06-15

**Authors:** Guanwen Wang, Chunhua Feng

**Affiliations:** 1 The Key Lab of Pollution Control and Ecosystem Restoration in Industry Clusters, Ministry of Education, School of Environment and Energy, South China University of Technology, Guangzhou 510006, China; w.guanwen@mail.scut.edu.cn; 2 Guangdong Provincial Engineering and Technology Research Center for Environmental Risk Prevention and Emergency Disposal, South China University of Technology, Guangzhou 510006, China

**Keywords:** electropolymerization, conducting polymer, electron mediator, anode modification, microbial fuel cell, confocal laser scanning microscopy

## Abstract

Here we reported the use of electropolymerization to achieve the transformation of aqueous hydroquinone to solid-phase polyhydroquinone (PHQ) with pseudocapacitive characteristics, and the application of this redox-active product to shuttle electron transfer in the anode system of a microbial fuel cell (MFC). The microscopic and spectroscopic results showed that the treatment of the graphite felt (GF) substrate with acids was effective in improving the amounts of surface-bound oxygen-containing groups, enabling better adhesion of PHQ onto the GF surfaces. The electrochemical measurements indicated that the resulting PHQ–AGF (acid treated GF) possessed high pseudocapacitance due to the fast and reversible redox cycling between hydroquinone and benzoquinone. The MFC equipped with the PHQ–AGF anode achieved a maximum power density of 633.6 mW m^−2^, which was much higher than 368.2, 228.8, and 119.7 mW m^−2^ corresponding to the MFC with the reference PHQ–GF, AGF, and GF anodes, respectively. The increase in the power performance was attributed to the incorporation of the redox-active PHQ abundant in C–OH and C=O groups that were beneficial to the increased extracellular electron transfer and enhanced bacterial adhesion on the anode.

## 1. Introduction

Hydroquinone and benzoquinone are well-defined aqueous redox couples that are susceptible to fast and reversible redox cycling and acid-base transformations. This redox feature allows them to be capable of reversibly transferring electrons and protons, and hence they have a wide range of applications including electrode materials for supercapacitors [1,2], mediators for biological processes [3,4], and catalysts for electrochemical reactions [5]. For example, the introduction of hydroquinone and benzoquinone couple into polyaniline significantly improved the pseudocapacitance and cycling stability due to their excellent electrochemical reversibility acting as mediating agents [1]. Similarly, it was reported that the polypyrrole electrode doped with benzoquinone groups exhibited much higher specific capacitance than the updoped reference electrode [2]. In addition, previous studies [6,7] have shown that the quinone group-functionalized electrodes were demonstrated to facilitate extracellular electron transfer from the bacterial cell to the electrode surface, and hence increased the anode performance and electricity generation of microbial fuel cells (MFCs), a promising technology extracting energy from wastewaters [6,7].

Motivated by hydroquinone and benzoquinone’s outstanding pseudocapacitive performance with fast electron transfer capabilities, here we propose a strategy to convert the aqueous hydroquinone into the solid redox-active polymer (i.e., polyhydroquinone, PHQ) and further utilize the solid film as a pseudocapacitive material effective in accelerating bioelectrochemical reactions of MFCs. A simple electropolymerization (i.e., electrooxidation) approach was adopted for hydroquinone recovery from aqueous solution, leading to the formation of redox-reactive PHQ. The graphite felt (GF) was used as the substrate to sustain the electropolymerization of hydroquinone due to its high surface area and good electric conductivity. Pretreatment of GF with mixed acids (denoted as AGF) was performed to increase surface oxygen functional groups, which were expected to be advantageous for the adhesion of PHQ. The anode performance was compared in terms of voltage and power output when PHQ was immobilized on the GF and AGF surfaces. The morphological and electrochemical characterizations of the inoculated anodes were conducted to monitor the bacterial adhesion and investigate the extracellular electron transfer.

## 2. Experimental Methods

### 2.1. Preparation of PHQ–GF and PHQ–AGF Electrodes and Electrochemical Tests 

The starting bare GF (Sanye Co., Ltd., Beijing, China) with dimensions of 3.0 cm × 2.0 cm × 0.5 cm was cleaned in a hot H_2_O_2_ (10%, 90 °C) solution for 3 h, followed by a thorough rinse with deionized water and drying at 60 °C. The AGF was obtained by immersing the GF into the mixed solution of sulfuric acid and nitric acid (volume ratio 3:1) for 1 h at 60 °C. The AGF was then repeatedly washed with deionized water until the pH value of the washed water reached 7.0. A Ti wire was folded to string the felts and twisted together to connect the external circuit. The room-temperature electropolymerization was performed with a CHI660e electrochemical potentiostat (CH Instruments, Chenhua Co., Shanghai, China) in a solution containing 0.1 M phosphate buffer solution (PBS, pH 7.0) and 1 mM hydroquinone. The three-electrode system was used for the electrooxidation of hydroquinone with the GF (or AGF), saturated calomel electrode (SCE), and Pt mesh as the working electrode, reference electrode, and counter electrode, respectively. The applied anodic potential was 2.0 V, an optimal value to obtain over-oxidized PHQ according to previous reports [3,8]. The thickness of the resulting PHQ–GF and PHQ–AGF composite films was controlled by maintaining the total applied charge at 3 C cm^−2^ (normalized to the projected electrode surface area). The reference GF and AGF electrodes were also prepared by exposure of the carbon electrodes to 2.0 V in the absence of hydroquinone, to investigate the contribution of the surface electrochemical activation to the capacitive currents. The time period applied to the electrode was controlled at the same value as that related to the presence of hydroquinone. Prior to further characterizations and uses, these samples were thoroughly washed with deionized water and air-dried at room temperature. 

Characterizations on the electrochemical capacitance of the GF, AGF, PHQ–GF, and PHQ–AGF electrodes were conducted by cyclic voltammetry (CV) and galvanostatic charge-discharge (GCD) tests using the same three-electrode system in a 0.1 M PBS solution (pH 7.0). The CV scans were conducted between −0.4 and 0.6 V at various scan rates (5–100 mV s^−1^). The GCD measurements were undertaken at a current load of 0.08 mA cm^−2^ within the potential window from −0.1 to 0.4 V. For non-linear GCD plots, the area specific capacitance was calculated according to the following equation [9]:

(1) $$C_{S}=\frac{I/S\int Udt}{\frac{U^{2}}{2}|\begin{array}{c} U_{f}\\ U_{i} \end{array}}=\frac{2S_{dis}I/S}{U^{2}|\begin{array}{c} U_{f}\\ U_{i} \end{array}}$$

where *C_S_* is the specific capacitance (F cm^−2^); *I*, *t*, *S*, and *S_dis_* represent the discharge current (A), discharge time (s), projected electrode surface area (cm^2^), and enclosed area of the discharge curve and coordinate axis, respectively; *U* (V) is the potential with initial and final values of *U_i_* and *U_f_*, respectively.

### 2.2. MFC Operation and Performance Tests

A dual-chamber MFC separated by a cation exchange membrane (Zhejiang Qianqiu Group Co., Ltd., Linan, China) was constructed according to the procedures described previously [10]. The reactors were made of polycarbonate and the effective volume of each chamber was 25 mL. The anode was the as-prepared PHQ–AGF, and the cathode was the unmodified GF (3 cm × 2 cm × 0.5 cm). For comparisons, the GF, AGF, PHQ–GF were used the reference anodes. Shewanella oneidensis MR-1 purchased from ATCC (700550) (American Type Culture Collection, Manassas, VA, USA) was used as the electrochemically active bacteria in the anode chamber. This species was cultured in LB medium (10 g L^−1^ peptone, 5 g L^−1^ yeast extract, and 5 g L^−1^ NaCl) at 30 °C. For MFC inoculation, one milliliter of the bacterial culture was pipetted into the sterilized anode chamber. The growth medium added to the anode chamber was composed of 20 mM lactate and 0.1 M PBS-based nutrient solution (pH 7.0) consisting of 5.84 g L^−1^ NaCl, 0.10 g L^−1^ KCl, 0.25 g L^−1^ NH_4_Cl, 10 mL L^−1^ of vitamin solution, and 10 mL L^−1^ of mineral solution [11]. The PBS solution (0.1 M, pH 7.0) with 50 mM potassium ferricyanide was added to the cathode chamber. Cell voltages were recorded every 2 min by a 16-channel voltage collection instrument (AD8223, Beijing, China) under the conditions of a 500 Ω external resistance and a controlled temperature of 30 °C.

It has been previously identified that the use of pseudocapacitive anode material can induce the transient power effect when the traditional varying circuit resistance (VCR) or linear sweep voltammetry (LSV) method [12] was used to determine the power performance. The power density curves were thus obtained by the fed-batch cycle test, during which the maximum sustainable potential over the complete cycle was recorded using a single resistor varying from open-circuit, 2000, 1000, 500, 250, 100, 50, to 25 Ω. The maximum sustainable potential recorded at each resistor typically sustained for 2 to 24 h depending on the total length of the cycle. The changes in the anode potential were measured by inserting a sterilized SCE into the anode chamber. The cell potential and the anode potential were recorded by the UT-805A desktop multimeter (ShenZhenUni-Trend Group Limited, Shenzhen, China) that connects to a computer. Current density (*I*, A m^−2^) and power density (*P*, mW m^−2^) were calculated according to Equations (2) and (3), respectively. Both *I* and *P* were normalized to the projected area of the anode surface.

(2) $$I=\frac{V}{R}$$

(3) P=V×I

where *R* is the resistance (Ω); *V* is the cell potential (V). To test reproducibility, repeated experiments for each treatment were conducted. The data presented below originated from a representative experiment if the results of triplicate experiments showed negligible difference; otherwise, the mean value of triplicate experiments was presented, with error bars indicating the standard deviations.

### 2.3. Physical and Electrochemical Characterizations

Surface morphologies of the electrodes before and after inoculation were characterized by scanning electron microscopy (SEM) with a Merlin electron microscope (Carl Zeiss AG, Oberkochen, Germany). The stabilization of the anode biofilm was conducted according to the previous procedures [10]. The confocal laser scanning microscopy (CLSM) tests were performed to visually illustrate the biofilms on the four electrodes. For pretreatment, a sample (0.5 cm × 0.5 cm) was sliced from the graphite felt surface, followed by flushing with sterilized PBS to remove loosely attached planktonic cells. These samples were then stained with the LIVE/DEAD^®^ BacLight™ Bacterial Viability Kit (for microscopy and quantitative assays) based on the manufacturer’s instructions. A Leica CLSM microscope (TCS SP8, Leica Microsystems, Wetzlar, Germany) was used for microscopic observations. Labeled cells were visualized and z-stacks were captured. 

The surface composition of all the samples was determined by X-ray photoelectron spectroscopy (XPS, Thermo Fisher Scientific, Waltham, MA, USA) with Al-Kα radiation (*h* = 1486.6 eV) and an X-ray power of 150 W. Fitting of the XPS peaks into different components was performed using the XPSPEAK41 software. The oxygen-containing groups available in different samples were qualitatively identified by Fourier transform infrared spectroscopy (FT-IR, Thermo Fisher SCIENTIFIC Nicolet IS10, Thermo Fisher, Waltham, MA, USA) with KBr pellets. 

The measurements of electrochemical impedance spectra (EIS) (CH Instruments, Chenhua Co., Shanghai, China) with respect to different inoculated anodes were recorded at the open circuit potential. The frequency range was from 100 to 0.01 Hz and the sinusoidal excitation signal was 10 mV. The measurements were performed in a three-electrode mode with the anode as the working electrode, a sterilized SCE inserted into the anode chamber as the reference electrode, and the cathode as the counter electrode. 

## 3. Results and Discussion

### 3.1. Electrochemical Capacitance Performance of the Composite Films

**Figure 1:**
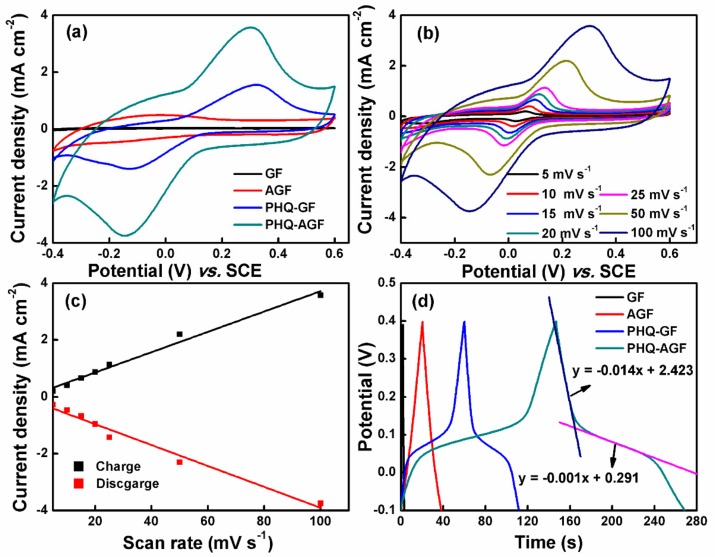
(**a**) CV profiles of the GF, AGF, PHQ–GF, and PHQ–AGF electrodes at a scan rate of 100 mV s^−1^; (**b**) CV profiles of the PHQ–AGF electrode at the scan rate ranging from 5 to 100 mV s^−1^; (**c**) Plot of Faradaic peak currents vs. scan rates derived from the CVs of the PHQ–AGF electrode; (**d**) GCD profiles of the GF, AGF, PHQ–GF, and PHQ–AGF electrodes at a current load of 0.08 mA cm^−2^.

The electrochemical capacitance performance of the PHQ–AGF composite electrodes and the reference GF, AGF, and PHQ–GF electrodes were evaluated and compared using the CV and GCD measurements. These measurements were performed in a three-electrode electrochemical cell containing 0.1 M PBS (pH, 7.0), an SCE reference electrode, and a Pt mesh counter electrode. Figure 1a shows the voltammograms of the four electrodes at a scan rate of 100 mV s^−1^. In comparison to the pristine GF, the AGF exhibited much higher current density, indicating increased capacitance. The CV curve of the AGF electrode revealed a weak and broad pair of redox peaks at −0.2–0 V, ascribed to the redox behavior of oxygen-containing functional groups on the surface [13,14]. This result reflects that the acid treatment is effective in increasing the Faradaic response due to the incorporation of oxygen-containing functional groups. Further increase in the current density was clearly visible in the CV curves of the PHQ–GF and PHQ–AGF composite electrodes, with the latter showing more pronounced increment. The distinctly observed redox pair at a mid-peak potential of 0.1 V were attributed to the reaction between polyhydroquinone and polybenzoquinone [3,8,15]. It should be noted that the electrochemical oxidation of the carbon at 2.0 V in the absence of hydroquinone can also increase the capacitive currents; nevertheless, the values of these currents were significantly lower than those obtained from the solutions with hydroquinone, and no redox peaks were observed (data not shown).

It can be concluded from Figure 1a that (i) the PHQ is successfully deposited on the GF surface and contributed to the large part of pseudocapacitance and (ii) the AGF surface is beneficial to the adhesion of PHQ. Figure 1b depicts the CV curves of the PHQ–AGF composite electrode at different scan rates (5–100 mV s^−1^). The noticeable increase in the current density with the increasing scan rate was indicative of the good rate ability of the PHQ–AGF electrode. A linear dependence of the Faradaic peak currents on scan rates was revealed in Figure 1c. This is characteristic of a surface-confined charge transfer process [16], which indicates that the surface-bound hydroquinone and benzoquinone couple are responsible for the pseudocapacitive charging/discharging. Similar behavior has also been reported for lignin materials [17,18] containing redox-active quinine groups, which deliver high pseudocapacitance. 

The GCD measurement is frequently used for the accurate determination of specific capacitance. As shown in Figure 1d, the remarkable difference in the discharge time between the four electrodes was recognized. The PHQ–AGF electrode displayed the longest discharge time, indicating the highest capacitance in accordance with the CV results. The specific capacitance of the PHQ–AGF electrode was calculated to be 129.4 F m^−2^, which was 2.5, 4.9, and 61.6 times larger than that obtained from the PHQ–GF electrode, the AGF electrode, and the GF electrode, respectively. It was distinctly observed that there were two potential slopes in the discharge curves of the PHQ–AGF and PHQ–GF electrodes. Similar phenomena were observed in previous reports concerning the integration of the pseudocapacitance with the double-layer capacitance in one hybrid material [17,18]. According to the CV results, the potential variation ranging from −0.01 to 0.12 V was considered to be the pseudocapacitive behavior of PHQ, having a slope of −0.001. This slope value was much higher than that (−0.014) related to other potential ranges, in which the double-layer capacitance of AGF dominated. Thus it could be assumed that the highest specific capacitance of the PHQ–AGF electrode is mainly attributed to the pseudocapacitance arising from the redox chemistry of PHQ.

### 3.2. Analysis of Oxygen-Containing Functional Groups

**Figure 2:**
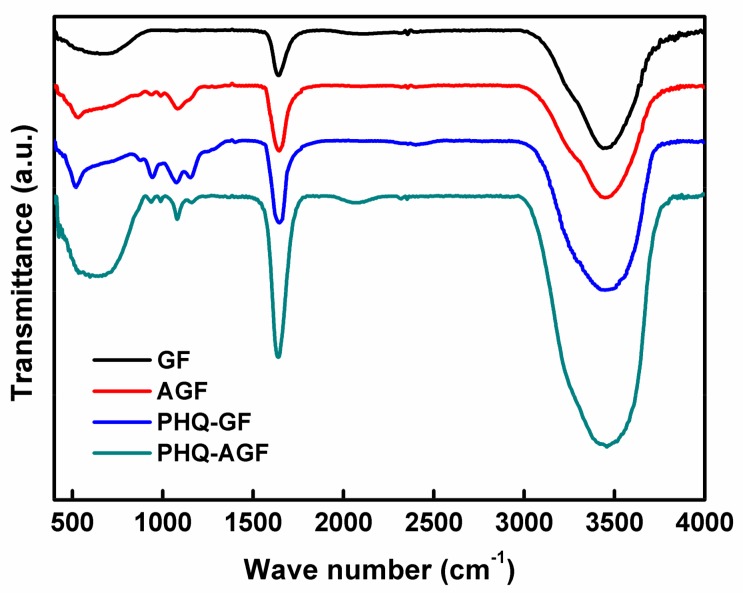
FT-IR spectra of the GF, AGF, PHQ–GF, and PHQ–AGF samples.

FT-IR is a frequently used technique to identify functional groups available in carbon materials, giving information of structural changes as a result of different treatments or modifications. Figure 2 depicts the FT-IR spectra of GF, AFG, PHQ–GF, and PHQ–AGF samples. It was clearly seen that the treatment of GF with HNO_3_+H_2_SO_4_ resulted in an increase in the intensity of the absorption band at 1636 cm^−1^ corresponding to the C=O stretching vibration [19,20,21], and a newly appeared absorption band at 1079 cm^−1^ due to the C–O stretching vibration [20,22]. This result is consistent with those reported in previous studies [13,14,19] concluding that acid treatment of carbon materials can introduce oxygen-containing functional groups such as hydroxyl and carboxylic groups. The deposition of PHQ on the GF or AGF surfaces enhanced the intensities of the C=O and C–O bands, characteristic of quinone and quinol groups, respectively [20,21]. In addition, new absorption bands were observed at 937 cm^−1^ likely attributed to the C–O–C group [23] and 1150 cm^−1^ likely associated with the C–O band in the carboxylic group [22]. The intensity of the band at 3458 cm^−1^ representing –OH stretching vibration [20] also increased. These enhancing effects were more pronounced in the PHQ–AGF sample compared to the PHQ–GF sample. The observations in Figure 2 evidence that electropolymerized PHQ onto the AGF surfaces significantly increase the amounts of oxygen-containing groups; the oxygen atoms in the PHQ should be present in the form of hydroxyl, carbonyl, and carboxylic groups.

**Figure 3:**
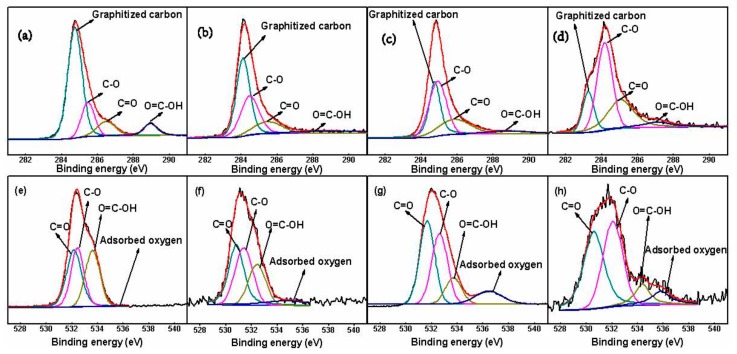
High-resolution spectra and fitting curves of the (**a**) C1s peak, GF; (**b**) C1s, AGF; (**c**) C1s, PHQ–GF; (**d**) C1s, PHQ–AGF; (**e**) O1s peak, GF; (**f**) O1s, AGF; (**g**) O1s, PHQ–GF; and (**h**) O1s, PHQ–AGF.

XPS is also a powerful technique to monitor the surface atomic content and surface chemistry of the carbon materials. The overall surface oxygen content with respect to different samples followed the ascending order: GF (18.6 at %) < AGF (39.6 at %) < PHQ–GF (46.5 at %) < PHQ–AGF (54.1 at %). Figure 3 shows the high-resolution C1s and O1s XPS peaks of GF, AFG, PHQ–GF, and PHQ–AGF samples. The deconvolution of the C1s spectra was fitted with four components [13,24] at 283.3–284.7, 285.2–285.5, 284.9–286.5, and 287.0–288.9 eV, corresponding to graphitized carbon, C–O, C=O, and O=C–OH groups, respectively. The O1s spectra can be deconvoluted into four peaks [24]: Peak 1 (530.8–532.2 eV), C=O group; peak 2 (531.4–532.6 eV), C–O group; peak 3 (532.5–533.7 eV), O=C–OH group; and peak 4 (534.7–536.4 eV), adsorbed oxygen. Table 1 summarizes the relative content of each component deconvoluted from the C1s and O1s peaks. The acid-treated sample possessed higher amounts of oxygen-containing groups as compared to the untreated sample. Further remarkable increase in the relative amounts of these functional groups was revealed in the PHQ modified samples, particularly for the PHQ–AGF sample. For example, the relative contents of C–O, C=O obtained from C1s deconvolution in the PHQ–AGF sample were 45.20% and 30.49%, respectively, higher than those relative to other samples. These results agree well with the FT-IR results.

### 3.3. Proposed Mechanism of Hydroquinone Electropolymerization on the Graphite Felt Surface

The mechanism of hydroquinone electropolymerized to PHQ is explained based on the literature data [15,25] and the aforementioned electrochemical and physical characterization results. Upon the application of an external electric field, the hydroquinone is first oxidized to the protonated form, with one hydroxyl group within the benzene ring oxidized to the cation radical, and the other one remaining unoxidized. This cation functions as an active species that electrophilically reacts with the non-protonated hydroquinone, leading to the formation of bipolymer with deprotonation. Owing to the lower oxidation potential of the bipolymer than that of the monomer, it can be further oxidized to its cation radical in the presence of the electric field, so that the propagation reaction occurs. The solid PHQ is thus formed and attached to the substrate, and the electropolymerization process is shown according to the following pathways [15,25].
(4)
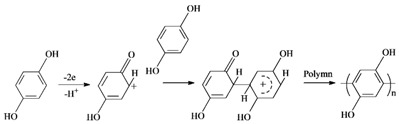


The resulting PHQ interacted with the GF surface via π–π stacking [26] and hydrogen bonding [27,28]. The hydrogen bonding interaction between the hydroxyl groups present in the PHQ and the oxygen-containing functional groups available in the GF results in the stabilization of the PHQ. More importantly, the increased amounts of oxygen groups on the AGF surface thus increasingly favor the adhesion of the PHQ. This PHQ on the AGF surface formed via electropolymerization exhibits the pronounced redox characteristics owing to the two electron-two proton charge transfer process between polyhydroquinone and polybenzoquinone [15], which was represented in the following reaction.
(5)
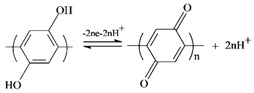


### 3.4. MFC Power Performance 

**Figure 4:**
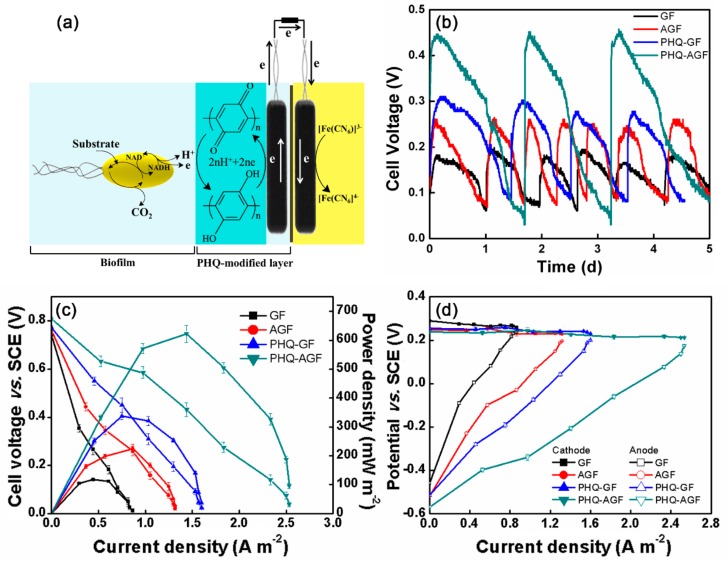
(**a**) Electron transfer pathway mediated by the PHQ-modified anode in a MFC. (**b**) Reproducible cell voltages of MFCs at a 500 Ω external resistor. (**c**) Cell polarization and power density curves of MFCs equipped with different anodes. (**d**) Cathode and anode polarization curves of MFCs equipped with different anodes.

The resulting PHQ–AGF composites are demonstrated to be a hybrid pseudocapacitive material combining electrochemical double-layer capacitance (EDLC) arising from GF and pseudocapacitance attributed to the Faradic reaction of PHQ. We have previously shown that the electrode materials beneficial for the increase in capacitance can also be effective in increasing the anode performance of MFCs [12], because the favorable characteristics such as high electrical conductivity, large surface area, and good interaction between the electrode and the electrolyte are desirable to both the supercapacitors and MFCs. This view has also been demonstrated by other researchers using hybrid pseudocapacitive materials such as manganese oxide@carbon felt [29,30], manganese oxide@polyaniline [31] and polypyrrole [32], manganese ferrite@polyaniline [33], titanium dioxide@nanocarbons [34], and graphene@graphite [35], which are able to facilitate electron transfer from the microorganisms to the anode and thus improve the anode performance. Here we examined the feasibility of using the PHQ–AGF composites as the MFC anode materials, in which PHQ functioned as an electron shuttle playing an important role in extracellular electron transfer. Figure 4a illustrates the electron transfer pathway mediated by the fast and reversible reaction between polyhydroquinone and polybenzoquinone. The redox potential of the hydroquinone and benzoquinone couple under the neutral condition is 0.10 V (as illustrated in Figure 1a), larger than −0.45 V related to the redox potential of Shewanella using lactate as the electron donor [36], which thus thermodynamically guarantees that benzoquinone can function as the electron acceptor driving lactate oxidation in the presence of Shewanella oneidensis MR-1. The electrons stored in hydroquinone can be further transferred through the external circuit to the terminal electron acceptors in the cathode, resulting in the reduction of ferricyanide to ferrocyanide. 

Power performance of the MFC equipped with the PHQ–AGF anode was evaluated and compared with that of the reference MFCs with the PHQ–GF, AGF, and GF anodes. Figure 4b shows the reproducible cell voltages of four MFCs at a 500 Ω external resistor. It was noticeable that the value of voltage produced in the MFCs followed the order: the PHQ–AGF anode (0.45 V) > the PHQ–GF anode (0.31 V) > the AGF anode (0.25 V) > the GF anode (0.18 V), highly consistent with the decreasing trend with respect to their capacitances. Figure 4c displays the power density curves, in which the variations in the power density as a function of the current density were plotted. Similarly, the PHQ–AGF anode achieved the highest maximum power density (633.6 mW m^−2^), increased by 1.8, 2.8, and 5.3 times as compared to the PHQ–GF, AGF, and GF anodes, respectively. It was evident from Figure 4d that the differences in the power performance predominantly stemmed from the variations in the anode performance. The cathode potentials of all MFCs exhibited negligible variations. In contrast, at each fixed current density, the PHQ–AGF anode had the lowest and most negative anode potential compared to other reference anodes, indicating the smallest polarization and the best anode performance. All the results in Figure 4 confirm that the hybrid PHQ–GF pseudocapacitive material can successfully boost the power performance of MFCs, when it is used as the anode material.

### 3.5. Morphological and Electrochemical Characterizations of the Inoculated Anodes

**Figure 5:**
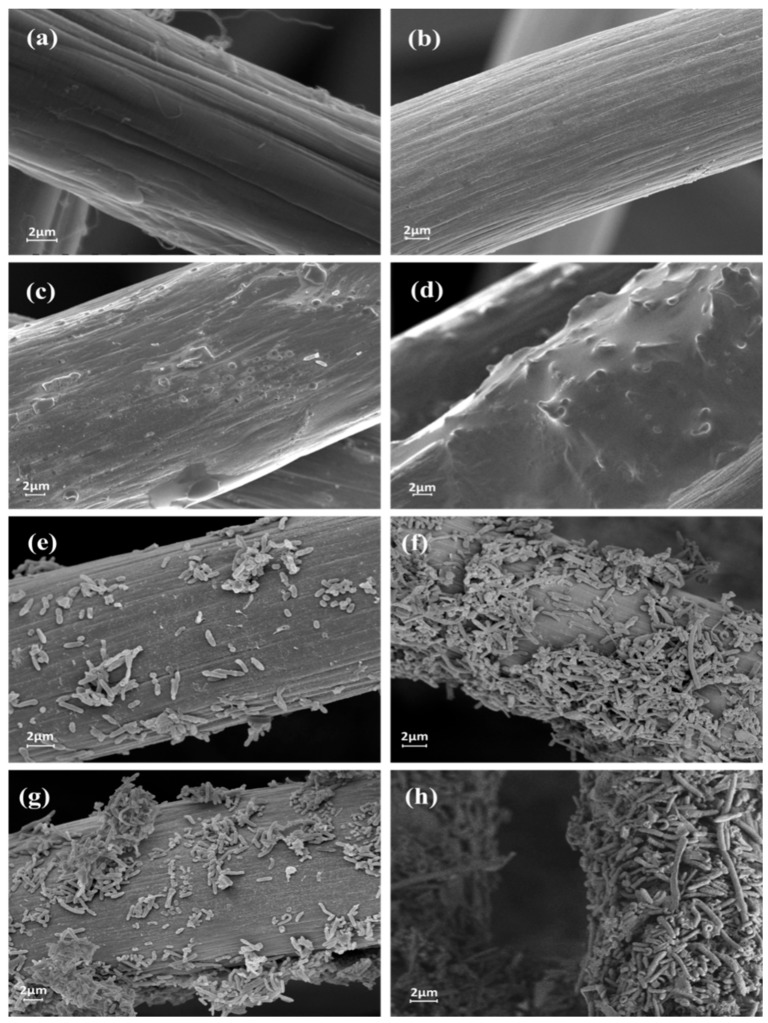
SEM images of the fresh (**a**) GF, (**b**) AGF, (**c**) PHQ–GF, (**d**) PHQ–AGF samples, and the (**e**) GF, (**f**) AGF, (**g**) PHQ–GF, (**h**) PHQ–AGF anodes inoculated with Shewanella oneidensis MR-1.

Figure 5 presents the SEM images of the four electrode samples before and after inoculation with the bacteria. Comparison between Figure 5a,b indicated that the treatment of GF with strong acids increased surface roughness without damaging its structure. Electropolymerization of hydroquinone onto the GF surface led to the formation of composite films (Figure 5c,d). It was apparent that more dense films were formed on the AGF surface than the GF surface, reflecting that the AGF rich in oxygen-containing functional groups favored the adhesion of PHQ possibly via the π–π stacking and hydrogen bonding. Figure 5e–h clearly indicates the variations in the amounts of bacteria attached on the anode surfaces, depending on their surface properties. Only scattered bacteria were grown on the GF and AGF surfaces, nevertheless, the AGF anode was beneficial to the higher numbers of bacteria attached. Bacteria developed biofilms on the PHQ-modified anode surfaces. It was observable that parts of the biofilms on the PHQ–GF surface were peered off. In contrast, the PHQ–AGF surface was favorable for the bacteria to form strongly-attached biofilms.

**Figure 6:**
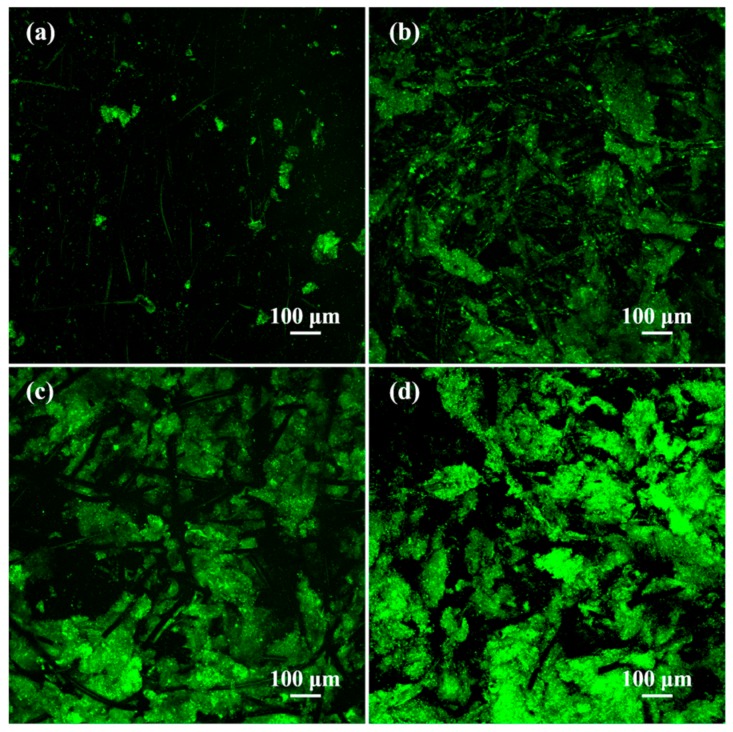
CLSM images of the biofilms on the (**a**) GF, (**b**) AGF, (**c**) PHQ–GF, (**d**) PHQ–AGF anodes.

Figure 6 shows and compares the CLSM images of the biofilms obtained from different anodes, with metabolically active (green) cells displayed. It was noticeable that lower numbers of living cells appeared in the untreated GF surface, while their amounts increased as a result of the treatment of GF with acids. The modification of GF with PHQ also resulted in more living cells attached to the anode surface. In addition, the biofilms on the PHQ–AGF surface showed the maximum thickness and numbers of living bacteria compared to those on other samples. These observations with a broad outline of the biofilm structure were in good agreement with the SEM results. These results demonstrate that the amounts of bacteria attached on the surfaces are highly sensitive to the surface chemistry of the anodes, confirming with previous findings [37,38,39] that the increased amounts of oxygen-containing groups (i.e., C–OH and C=O groups) substantially promote bacterial attachment. The possible reasons include that (i) they increase the surface wettability more accessible to the hydrophilic membrane of bacteria [38] and (ii) they can directly interact with the bacteria by forming chemical bonding (i.e., hydrogen and/or peptide bonding) with –OH and/or –NH_2_ groups on the bacteria surface [38,40,41].

**Figure 7:**
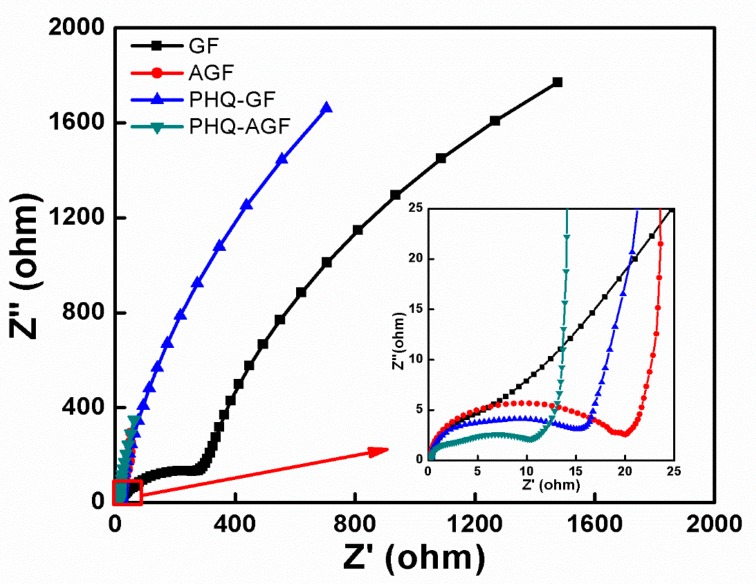
EIS curves (in the form of Nyquist plots) of the different anodes after inoculation.

The EIS technique has been frequently used to characterize the variations in the anode resistance as a function of different anode materials [6,12,39]. In general, the high-frequency intercept with the *x*-axis in the Nyquist plot indicates the ohmic resistance; and the diameter of the following semicircle represents the charge transfer resistance. As illustrated in Figure 7, all the inoculated anodes showed little difference in the ohmic resistance. The charge transfer resistance of the anode with biofilms was, however, highly relied on the anode materials. The PHQ–AGF anode had the smallest charge transfer resistance, indicating the fastest electron transfer between the microbes and the anode. The decreasing tread of this resistance value was found to be PHQ–AGF > PHQ–GF > AGF > GF, in accordance with the trend of their anode performance. The EIS results combined with the morphological data (SEM and CLSM) as well as the power performance variations confirm that the PHQ-modified anode surface favors the increased number of bacteria adhered, thus enabling a higher electron transfer rate and enhanced bioelectricity generation.

## 4. Conclusions

We demonstrated that electropolymerization of aqueous hydroquinone onto the GF surface resulted in the formation of the PHQ-modified GF composite film, a hybrid material showing increased pseudocapacitance and that is effective in promoting anodic bioelectrochemical reactions and thus the overall output of MFCs. The treatment of GF with strong mixed acids (HNO_3_ + H_2_SO_4_) significantly improved the amounts of oxygen-containing groups, which were beneficial to the attachment of PHQ. The characterizations by SEM, FT-IR, and XPS provided evidence that PHQ was effectively attached on the AGF surfaces and that these surfaces were substantially rich in the C–OH and C=O groups. The increased pseudocapacitance due to the incorporation of PHQ in the as-prepared composite electrodes was verified by the electrochemical CV and GCD measurements. By taking advantage of the unique pseudocapacitance behavior, we used the resulting PHQ–AGF as an anode material in the MFC. The results showed that the PHQ–AGF anode yielded a maximum power density of 633.6 mW cm^−2^, increased by 1.8, 2.8, and 5.3 times as compared to that obtained from the reference PHQ–GF, AGF, and GF anodes. As evidenced from the SEM, CLSM, and EIS results, the role of PHQ in facilitating extracellular electron transfer and promoting adhesion of bacteria was considered to be the main factor contributing to the increased anode performance and hence the MFC power performance. Taking into account that hydroquinone is one of the phenolic compounds widely found in the wastewater effluents of various industries such as coke, rubber, textiles, steel, petroleum refinery, dyes, pharmaceuticals, and cosmetics [42,43], this study might provide a useful method for the recovery of hydroquinone from wastewater streams. Future study needs to examine the possibility of immobilizing hydroquinone available in the real wastewaters.

## Figures and Tables

**Table 1:** Relative contents of functional groups deconvoluted from the C1s and O1s XPS peaks.

	C1s				O1s				
	Graphitized Carbon	C–O	C=O	O=C–OH		C=O	C–O	O=C–OH	Adsorbed Oxygen
GF	64.37%	17.40%	10.46%	7.77%		35.40%	31.00%	30.63%	2.98%
AGF	47.40%	31.95%	14.29%	6.36%		35.86%	32.34%	22.56%	9.25%
PHQ–GF	32.33%	40.94%	21.06%	5.67%		39.72%	35.35%	13.57%	11.36%
PHQ–AGF	16.51%	45.20%	30.49%	7.79%		43.16%	38.21%	10.06%	8.57%
